# Women Empowerment and Skilled Birth Attendants among Women in Rural Ghana

**DOI:** 10.1155/2021/9914027

**Published:** 2021-12-24

**Authors:** Kwamena Sekyi Dickson

**Affiliations:** Department of Population and Health, University of Cape Coast, Ghana

## Abstract

**Background:**

A critical public health issue is maternal mortality. Around 810 women die per day from pregnancy and childbirth, with approximately 99 percent of these deaths recorded in low-and middle-income countries (LMICs). In sub-Saharan Africa (SSA), more than half of these mortalities are registered. The situation is remarkably similar in Ghana, with maternal mortality standing at 319 deaths per 100,000 live births in 2015.

**Methods:**

Using data from 2014 Demographic and Health Surveys, the study examined the association between women empowerment and skilled birth attendance among women in rural Ghana.

**Results:**

Women with medium decision-making (OR = 0.75, CI = 0.61, 0.93), low knowledge level (OR = 0.55, CI = 0.40, 0.76), high acceptance of wife beating (OR = 0.68, CI = 0.51, 0.90), with less than 4 ANC visits (OR = 0.25, CI = 0.19, 0.32), whose partner had higher education (OR = 1.96, CI = 1.05, 3.64), and who had a big problem with the distance getting to the health facility (OR = 0.63, CI = 0.50, 0.78) had a significant association with skilled birth attendants. Decision-making power, women's knowledge level, acceptance of wife beating, antenatal care visit, partner's education, getting medical help for self, and distance to health facility were seen to have a significant association with skilled birth attendants among women in Ghana.

**Conclusion:**

Efforts to increase the current SBA should concentrate on the empowerment of women, male involvement in maternal health problems, women's education, and participation in the ANC. There is a need to review current policies, strategies, and services to improve maternal health conditions.

## 1. Background

A critical public health issue is maternal mortality. Around 810 women die per day from pregnancy and childbirth [1], with approximately 99 percent of these deaths recorded in low- and middle-income countries (LMICs) [2]. In sub-Saharan Africa (SSA), more than half of these mortalities are registered [3]. The situation is remarkably similar in Ghana, with maternal mortality standing at 319 deaths per 100,000 live births in 2015 [4]. Owing to the shattering consequences of maternal mortality, there has been an increasing need to reduce the incidence of maternal mortality particularly in SSA and specifically in Ghana. Hence, the United Nations in its 17 Sustainable Development Goals (SDGs) offered the blueprint to direct countries in their quest to minimize maternal mortality. SDG 3 is aimed at reducing the global MMR by 2030 to less than 70 per 100,000 live births [5].

Skilled birth attendants (SBA) are one of the essential instruments or techniques that have been postulated to substantially contribute to reducing maternal mortality. The WHO describes SBA as an accredited health professional who is competent in the skills needed to manage normal (uncomplicated) pregnancies and childbirth and to identify, manage, and refer complications in women and newborns, such as a midwife, doctor, or nurse [6]. The utilization of SBA has been found to avert 16-33 percent of maternal mortality [2]. Available evidence indicates that delivering at home results in high-risk pregnancy which can lead to complications such as haemorrhage and sepsis [7]. Therefore, delivering through SBA becomes a significant conduit for reducing pregnancy complications and maternal mortality.

Several studies have also shown that there are variations in the utilization of SBA across the rural-urban dichotomy, with women in the rural communities being the most disadvantaged. For example, Alemayehu and Mekonnen [8] recorded that SBA delivery was 7.2 times more likely to be used by urban-dwelling women compared to their rural counterparts. This low use of SBA by rural women has been related to long distances to health facilities, limited knowledge of the benefits of SBA delivery, long waiting times, and transport problems [9, 10]. Beyond these reasons, however, there is an increasing interest among rural-dwelling women in the link between women's empowerment and SBA.

The empowerment of women should be one of the aspects of maternal health as it has important effects on women's health-seeking behaviour. The empowerment of women involves topics such as economic participation, household decision-making power, and attitudes towards wife beating [3]. These composite factors that constitute women's empowerment may influence SBA. Moreover, empirical evidence from Nigeria [11] and Uganda [12] has demonstrated that women's empowerment significantly affects SBA. Despite the ability of women's empowerment in predicting the use of SBA, there is no empirical evidence of this in Ghana. Therefore, the connection between women's empowerment and SBA among women in rural Ghana was investigated in this paper.

## 2. Methods

### 2.1. Data Source

The research made use of data from the 2014 Ghana Demographic and Health Surveys (DHS). The DHS is a national representative study conducted in many low- and middle-income nations in Asia and Africa over a five-year period. By interviewing women during their reproductive age, it focuses on maternal and child health (15-49 years). In areas such as sampling, questionnaires, data collection, cleaning, coding, and analysis, the DHS follows standardized procedures which allow for comparability across countries. For this analysis, only women who had given birth in the five years before the survey and have been staying in the rural areas were included, which is a sample of 2,093.

### 2.2. Selection of Variables

#### 2.2.1. Outcome Variable

Skilled birth attendance was the principal outcome variable. The result variable was derived from the answer to the question “who assisted with the delivery?” Responses were classified as “1” and “0” by health personnel and other people. Health personnel included physicians, nurses, nurses/midwives, and auxiliary midwives; other individuals also included traditional birth attendants (TBAs), traditional health volunteers, community/village health volunteers, and neighbors/friends/relatives. Skilled birth attendance for this research referred to births assisted by a physician, nurse, auxiliary midwife, and nurse/midwife [13, 14].

#### 2.2.2. Explanatory Variable

Women empowerment was the main explanatory variable. The elements of women consist of (1) labour force (working, not working), (2) acceptance of wife beating (neglect of a child, burning of food, arguing with husband/partner, refusal to have sex with husband/partner, going out without permission), (3) decision-making power (this is measured by the respondents' health care, house earning and household purchase, and visiting family members), and (4) knowledge level (listening to radio, read newspaper/magazine, watching television, and educational level). This is in accordance with the methods of previous authors [15, 16]. Nine other explanatory variables were also used, namely, age, partner's level of education, number of antenatal care (ANC) visits, getting medical help for self, money needed for treatment, and distance to a health facility.

Age was captured as 15–19, 20–24, 25–29, 30–34, 35–39, 40–44, and 45–49. Women's partner's levels of education were classified as no education, primary, secondary, and higher education. The number of antenatal care (ANC) visits was captioned as less than four visits and four or more visits. Getting medical help for self: money needed for treatment and distance to a health facility were labeled as a big problem and not a big problem.

### 2.3. Statistical Analysis

There were descriptive and inferential analyses performed. Results on background features and the prevalence of skilled birth attendance were reported in the descriptive analysis. Two models were used for the inferential analysis. Model I looked at the relationship between the women empowerment variables and skilled birth attendants. Model II was used to explore the relationship between skilled birth attendance and women empowerment plus the background characteristics. Precisely, binary logistic regression was performed. With 95% confidence intervals, all results of the binary logistic analyses were presented as odds ratios (ORs) and confidence intervals (CIs). All analyses were done using version 14 of Stata.

#### 2.3.1. Model Fit and Specifications

The fitness of the models with the likelihood ratio (LR) test was assessed. The presence of multicollinearity between the independent variables was checked before fitting the models. The variance inflation factor (VIF) test revealed the absence of high multicollinearity between the variables (Mean VIF = 1.41, Minimum = 1.09, and Max VIF = 1.60).

## 3. Results

### 3.1. Background Characteristics and Skilled Birth Attendants

Women with high knowledge levels commonly had skilled birth attendants (72.5%). Six in ten women with high decision-making ability, low acceptance of wife beating, and participation in the labour had skilled birth attendants during delivery (see Table 1). Women whose partners had higher education (87.1%) usually had the services of skilled birth attendants during delivery. Six in ten women who did not have a big problem getting the money needed for health care and those who did not have a big problem with distance to the health facility utilized the services of skilled birth attendants during delivery (See Table 1).

### 3.2. Binary Logistic Regression of Skilled Birth Attendants among Rural Women in Ghana

Results for all the models were presented in Table 2. The final model II showed that decision-making, knowledge level, acceptance of wife beating, ANC visits, partner's education, getting medical help for self, and distance to health facility were seen to have a significant association with skilled birth attendants.

Women with medium decision-making (OR = 0.75, CI = 0.61, 0.93) were less likely to utilize the service of a skilled birth attendant during delivery compared to those with high decision-making. Women with low knowledge levels (OR = 0.55, CI = 0.40, 0.76) had a less likelihood of utilizing the service of a skilled birth attendant during delivery compared to those with medium knowledge level (see Table 2). Women with high acceptance of wife beating (OR = 0.68, CI = 0.51, 0.90) had lesser odds of utilizing the service of skilled birth attendants compared to those with low acceptance of wife beating. Women with less than 4 ANC visits (OR = 0.25, CI = 0.19, 0.32) had a less likelihood of utilizing the service of skilled birth attendants during delivery (see Table 2).

Women whose partner had higher education (OR = 1.96, CI =1.05, 3.64) had a higher likelihood of utilizing the service of skilled birth attendants during delivery compared to those whose partners had secondary education. Women who had a big problem with the distance getting to the health facility (OR = 0.63, CI = 0.50, 0.78) had a lesser likelihood of utilizing the service of a skilled birth attendant during delivery compared to those who did not see the distance to the health facility as a big problem (see Table 2).

## 4. Discussion

A higher number of prenatal consultations had a positive association for skilled birth attendants, low decision-making power, a lower level of education, distance to health facility being a big problem, and acceptance of wife beating had a negative association for skilled birth attendants. A higher partner education had a positive association for skilled birth attendants. The study noted that in rural Ghana, women with medium decision-making power are less likely to use the service of a trained birth attendant during delivery. This is consistent with earlier studies by Ameyaw and colleagues in 2016 [4] who found out that women with less decision-making autonomy had a lower tendency to deliver in the health facility. Similar results were also seen by Shimamoto and Gipson, [2], Fawole and Adeoye [17], Asweto et al. [18], and Say et al. [7]. The possible explanation may be as women are in the capacity to decide on their health and well-being, they make better maternal health care decision outcomes.

Women with low knowledge level were seen to have a lesser likelihood of utilizing the service of a skilled birth attendant during delivery in rural Ghana. Baatiema et al. [19], Shimamoto and Gipson [2], and Gitimu et al. [20] reported similar results that knowledge level has a significant association with a skilled birth attendant. When women have less knowledge level, they may not be aware of the complications and health risks of not delivering at the health facility. The more informed women are, the more likely they would choose to deliver at the health care facility.

Women with a high acceptance of wife beating were found to have a lower probability of using a qualified birth attendant's service during delivery in rural Ghana. This is in line with previous research [17, 21]. Women who made fewer than four visits to antenatal care had a lower risk of using a trained birth attendant's service during delivery in rural Ghana. Antenatal care is meant to be a precursor to childbirth for all women worldwide, and because of its proven gains in protecting maternal and infant health, the existing WHO recommendations mandate women to have a minimum of 4 ANC visits. This is in line with previous studies in Ghana [19]; Sierra Leone, Niger, and Mali [13]; and Kenya [20].

Women whose partners had a higher level of education had a higher probability of using the service of a trained birth attendant during delivery in rural Ghana. Training helps to create an educated decision for individuals. This result is in line with previous research by Tessema and Tesema [22], Saah et al. [23], and Dickson and Amu [14]. The potential reason would be that they are more likely to advise their partners to have health care delivery when women's partners have higher education and are well aware of the danger signs in pregnancy and the need to be accompanied by a trained birth attendant. In most sub-Saharan African countries, because of the patriarchal structure, men have a majority say in making health-related decisions and paying for bills, travel, and good nutrition. Women who had a major problem with the distance to health facilities had a lower probability of using a trained birth attendant's service during delivery in rural Ghana [14, 20, 24].

### 4.1. Strength and Limitations

The robust analytical and statistical strategy employed to enhance the reliability of our findings is the study's strength. In order to improve the study's replicability, we also supplied a full methodological protocol. The study's generalizability to all women in the reproductive age range in Sierra Leone is further enabled by the use of nationally representative data. Despite this, because DHS used a cross-sectional approach, we were unable to demonstrate causality between the numerous components in our investigation. Also, no data was available on this age group (10-14 years), and there can births in girls in this age group. Furthermore, responses were self-reported and there is a risk of recollection bias; therefore, the data should be interpreted with caution.

## 5. Conclusion

The study revealed that decision-making ability, level of women's awareness, acceptance of wife beating, antenatal care visit, partner education, receiving self-medical assistance, and distance to health facilities have been shown to have a significant association among women in Ghana with qualified birth attendants. Efforts to increase the current SBA should concentrate on the empowerment of women, male involvement in maternal health problems, women's education, and participation in the ANC. There is a need to review current policies, strategies, and services to improve maternal health conditions.

## Figures and Tables

**Table 1:** Background characteristics and skilled birth attendants.

Variable	Frequency (*n* = 2,093)	Percentage	Proportion of skilled birth attendant
Women empowerment			
*Decision-making ability power*			
Low	167	8.0	46.8
Medium	657	31.4	55.8
High	1,269	69.6	66.5
*Women's knowledge level*			
Low	214	10.2	36.6
Medium	1,087	52.0	58.5
High	792	37.8	72.5
*Labour force*			
Not working	313	15.0	62.9
Working	1,780	85.0	61.3
*Acceptance of wife beating*			
Low	1,439	68.7	66.0
Medium	364	17.4	58.1
High	290	13.9	44.0
Age			
15–19	55	2.6	68.7
20–24	345	16.5	61.4
25–29	533	25.4	64.3
30–34	454	21.8	56.6
35–39	413	20.0	64.1
40–44	209	10.0	63.0
45–49	84	4.0	51.2
ANC visit			
Less than 4	332	15.9	27.1
4 or more	1,761	84.1	68.1
Partner's educational level			
No education	654	31.2	44.4
Primary	302	14.5	61.6
Secondary	1,025	49.0	69.7
Higher	112	5.3	87.1
Getting medical for self: money needed for treatment			
Big problem	1,025	49.0	54.0
Not a big problem	1,068	51.0	68.8
Getting medical help for self: distance to a health facility			
Big problem	753	36.5	49.4
Not a big problem	1,330	63.5	68.6

**Table 2:** Binary logistic regression of skilled birth attendants among rural women in Ghana.

Variable	Model I odds ratio (confidence interval)	Model II odds ratio (confidence interval)
Women empowerment		
*Decision-making power*		
Low	0.62^∗∗^ (0.45, 0.85)	0.74 (0.53, 1.04)
Medium	0.76^∗∗^ (0.62, 0.93)	0.75^∗∗^ (0.61, 0.93)
High	Ref	Ref
*Women's knowledge level*		
Low	0.39^∗∗∗^ (0.29, 0.52)	0.55^∗∗∗^ (0.40, 0.76)
Medium	Ref	Ref
High	1.50^∗∗∗^ (1.21, 1.85)	1.01 (0.79, 1.28)
*Labour force*		
Not working	1.05 (0.81, 1.37)	1.03 (0.78, 1.38)
Working	Ref	Ref
*Acceptance of wife beating*		
Low	Ref	Ref
Medium	0.73^∗∗^ (0.57, 0.92)	0.89 (0.69, 1.15)
High	0.55^∗∗∗^ (0.42, 0.72)	0.68^∗∗^ (0.51, 0.90)
Age		
15–19		1.57 (0.83, 2.95)
20–24		1.18 (0.87, 1.61)
25–29		Ref
30–34		0.93 (0.71, 1.24)
35–39		1.04 (0.78, 1,40)
40–44		1.20 (0.84, 1.71)
45–49		0.75 (0.46, 1.22)
ANC visit		
Less than 4		0.25^∗∗∗^ (0.19, 0.32)
4 or more		Ref
*Partner's educational level*		
No education		0.55^∗∗∗^ (0.44, 0.70)
Primary		0.86 (0.64, 1.15)
Secondary		Ref
Higher		1.96^∗∗^ (1.05, 3.64)
Getting medical for self: money needed for treatment		
Big problem		Ref
Not a big problem		1.09 (0.88, 1.37)
Getting medical help for self: distance to a health facility		
Big problem		0.63^∗∗∗^ (0.50, 0.78)
Not a big problem		Ref

^∗^ *p* < 0.05, ^∗∗^*p* < 0.01, ^∗∗∗^*p* < 0.001. Ref: reference category.

## Data Availability

The datasets generated and/or analysed during the current study are available in the Measure DHS repository, https://dhsprogram.com/data/available-datasets.cfm, or The DHS Program—Available Datasets.

## Abbreviations

CI: Confidence interval
DHS: Demographic and health survey
LR: Likelihood ratio
SBA: Skilled birth attendants
SSA: Sub-Saharan Africa.
